# Longitudinal changes in the ALPS index and its clinical correlates in patients with basal ganglia hemorrhage

**DOI:** 10.3389/fneur.2026.1839634

**Published:** 2026-07-09

**Authors:** Zhaofeng Su, Jiajia Chen, Peng Wu, Rongjun Zhang, Xiaochi Yang, Nan Dong, Zhihui Fu

**Affiliations:** 1 Department of Radiology, Suzhou TCM Hospital Affiliated to Nanjing University of Chinese Medicine, Suzhou, China; 2 Philips Healthcare, Shanghai, China; 3 Department of Neurosurgery, Suzhou TCM Hospital Affiliated to Nanjing University of Chinese Medicine, Suzhou, China

**Keywords:** ALPS index, cerebral hemorrhage, longitudinal study, lymphatic system, neuroimaging

## Abstract

**Objective:**

To evaluate the robustness of Diffusion Tensor Image Analysis Along the Perivascular Space (DTI-ALPS) index, and to investigate its correlation with clinical characteristics in patients following basal ganglia hemorrhage.

**Methods:**

(1) Twenty-three age- and sex-matched healthy controls were enrolled and underwent DTI scans using both 15-direction and 32-direction sequences. The differences and consistency of ALPS index between the two protocols were compared. (2) A mixed cross-sectional and longitudinal study was conducted on 56 patients. Patients were divided into three groups by time since hemorrhage: ≤ 14 days, 3 months and 1 year. The ALPS index was calculated and compared among patient groups and against healthy controls. Correlations between the ALPS index with hematoma volume and the Brunnstrom recovery stage for the hand (BRS-H) were analyzed.

**Results:**

(1) Comparison of ALPS index calculated from 15-direction and 32-direction DTI data revealed significant differences in the left hemisphere (ALPS-L) between the two protocols, while no statistically significant difference was observed in the right hemisphere (ALPS-R) and the bilateral mean (ALPS-mean). The consistency between the two protocols was excellent across all groups (ICC = 0.959–0.981, *p* < 0.05). (2) ① ALPS index differed significantly among the three patient groups (*p* < 0.05). ALPS index for each group were as follows: Group A (*n* = 34): 1.24 ± 0.03; Group B (*n* = 12): 1.14 ± 0.04; Group C (*n* = 10): 1.18 ± 0.04. The ALPS index in all patient groups were significantly lower than in controls (*p* < 0.001). ② No significant correlation was found between the ALPS index and the BRS-H (*r* = 0.219, *p* > 0.05). ③ Overall, no significant correlation was observed between ALPS index and hematoma volume (*r* = −0.252, *p* > 0.05). However, stratified analysis showed a significant negative correlation in the ≤ 10 mL subgroup (*r* = −0.529, *p* < 0.05), but not in the > 10 mL subgroup (*r* = −0.091, *p* > 0.05).

**Conclusion:**

Basal ganglia hemorrhage is associated with impaired glymphatic function, with partial recovery after 3 months. The ALPS index showed a significant negative correlation with hematoma volume, supporting its sensitivity to hematoma-induced brain injury.

## 1 Introduction

Spontaneous intracerebral hemorrhage (ICH) refers to intraparenchymal bleeding caused by the spontaneous rupture of cerebral vessels due to non-traumatic factors. Supratentorial basal ganglia hemorrhage is most commonly attributed to hypertension (1). The basal ganglia serve as a critical motor integration center. The corticospinal tract traversing this region represents the largest descending motor pathway, primarily governing voluntary movements of the contralateral limbs, while the corticopontocerebellar tract is mainly involved in the fine motor control of the extremities (2). In the early stage of basal ganglia hemorrhage, the hematoma damages the fiber tracts through mechanical effects such as displacement and compression. As the disease progresses, the hematoma metabolic byproducts, and neuroinflammation further impair these fiber tracts through direct or indirect injury mechanisms. Due to the limited regenerative capacity of nerve fibers, along with axonal degeneration, demyelination, and glial scar formation that persist after hematoma absorption, neural signal transmission may be chronically impaired, leading to varying degrees of motor dysfunction that are often difficult to recover from (3). Despite ongoing advancements in medical care, the overall prognosis for these patients remains poor (4, 5). Moreover, survivors of ICH continue to face a range of sequelae, including delayed neurological injury, cardiac complications, and depression. Current management of basal ganglia hemorrhage is primarily conservative, as no evidence to date supports that surgical hematoma evacuation improves neurological outcomes (5). Therefore, in-depth exploration of imaging biomarkers associated with the pathophysiological processes of basal ganglia hemorrhage may not only facilitate the development of therapeutic strategies at the microstructural level but also provide crucial support for the objective quantitative assessment of treatment efficacy. Previous longitudinal studies on ICH have shown that neurological function scores correlate significantly with hematoma and perihematomal volumes, but not with parameters of microstructural integrity: magnetic susceptibility (*χ*), fractional anisotropy (FA), mean diffusivity (MD), or cerebral blood flow (CBF) (6). Given the aforementioned limitations, it is necessary to develop effective methods for assessing microstructural changes, thereby providing reliable imaging biomarkers for the evolution of ICH. To this end, we attempted to analyze the correlation between the glymphatic system (GS) and basal ganglia hemorrhage. The GS, discovered by Iliff et al. in 2012 (7), has elucidated a pathway for clearing metabolic waste generated in brain disorders such as stroke, Parkinson’s disease, and Alzheimer’s disease (8–11). This system consists of perivascular spaces (Virchow-Robin spaces) around arteries and veins, and astrocytic endfeet expressing aquaporin-4 (AQP-4) water channels. It is driven by cerebrospinal fluid (CSF) pressure gradients generated by arterial pulsations and respiratory movements. CSF from the subarachnoid space flows along arterial perivascular spaces into the brain parenchyma, and interstitial fluid, after completing material exchange, is absorbed into the systemic circulation via pathways including the arachnoid granulations (12). In recent years, diffusion tensor image analysis along the perivascular space (DTI-ALPS) has been increasingly applied for the noninvasive assessment of GS function (13). The ALPS method is based on the hypothesis that interstitial fluid movement in the white matter region at the level of the lateral ventricle body is parallel to the para-venous spaces and perpendicular to the lateral ventricular wall (and thus also perpendicular to the main orientations of white matter fibers: projection fibers running craniocaudally and association fibers running anterior-posteriorly). It uses a mathematical model to exclude the diffusion influence of these two major white matter fiber groups, to evaluate the minor diffusion component along the perivascular space (14). To date, there has been limited research on the association between the GS and ICH (9, 15).

The primary objective of this study was to investigate the longitudinal changes in the ALPS index following basal ganglia hemorrhage and its associations with clinical indicators. Given that patient data came from two different DTI protocols, we first assessed the robustness of the ALPS index to ensure cross-protocol comparability. We hypothesized that the ALPS index would decrease during the acute phase and gradually recover in subacute-to-chronic phases, and that lower ALPS index would correlate with larger hematoma volume and poorer motor function. Specifically, this study aimed to: (1) validate the robustness of the ALPS index between two diffusion protocols in healthy controls; (2) characterize longitudinal changes in the ALPS index across different post-ictal stages; and (3) explore correlations between the ALPS index and clinical indicators including hematoma volume and hand motor function assessed by the Brunnstrom Recovery Stage.

## 2 Materials and methods

### 2.1 Participants

**Figure 1:**
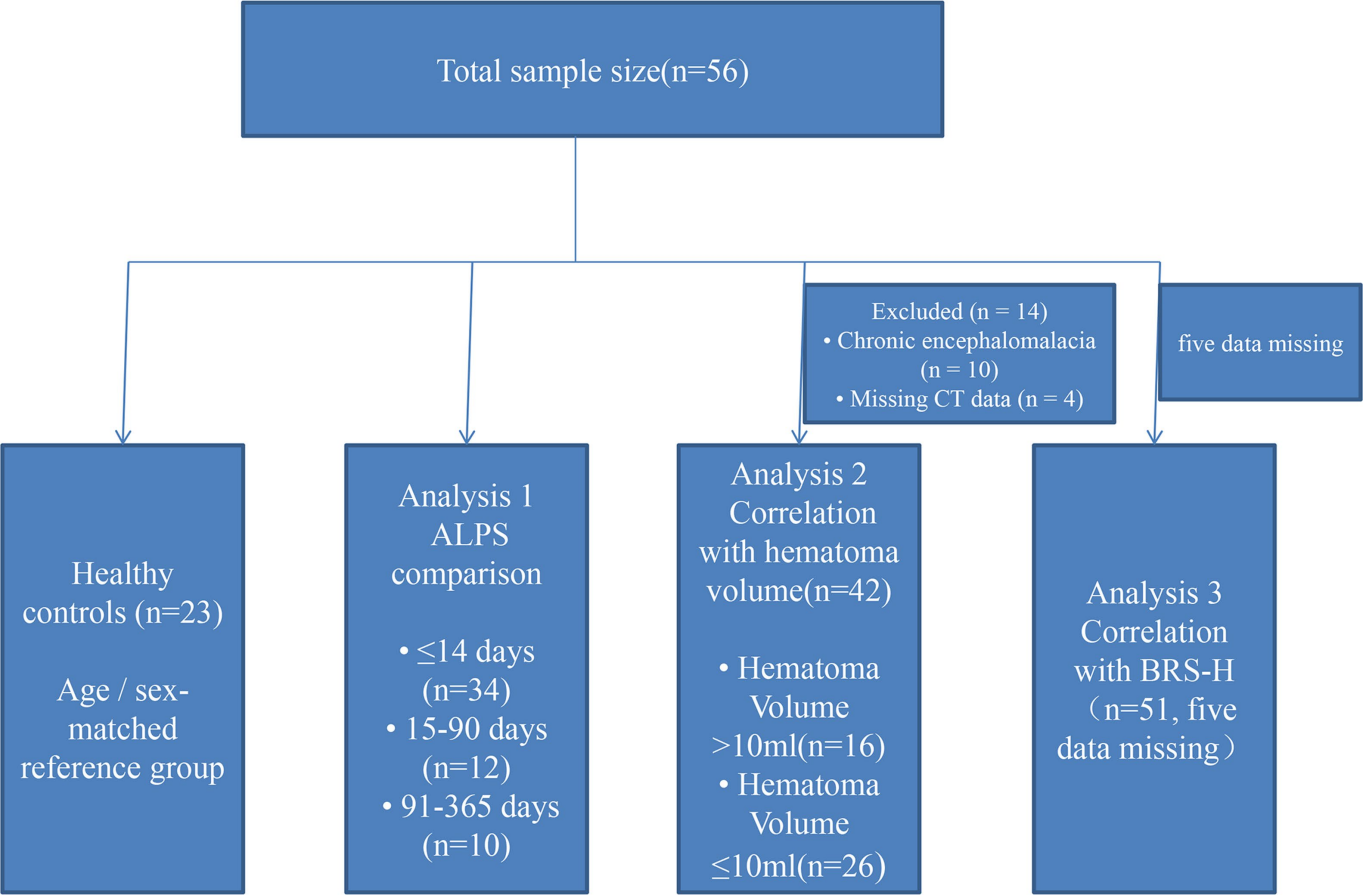
Flowchart of patient enrollment and study analyses.

This retrospective study employed a mixed design, primarily cross-sectional with a longitudinal subset (*n* = 6). The main analysis included 56 patients with basal ganglia hemorrhage who underwent MRI DTI scans between June 2022 and December 2025, while the longitudinal arm provided real-world follow-up data. All patients received conservative treatment. Inclusion criteria were: (1) supratentorial hemorrhagic lesion located to the basal ganglia; (2) absence of recent head trauma. Exclusion criteria were: (1) history of cognitive impairment or neuropsychiatric disorders; (2) secondary hemorrhage causes, such as hemorrhagic transformation of ischemic stroke, aneurysm, cavernous malformation, arteriovenous malformation, cerebral venous thrombosis, trauma, or tumor; (3) patients with extensive encephalomalacia or obvious chronic cerebral infarction. In addition, 23 age- and sex-matched healthy volunteers were recruited as controls. All partcipants were right-handed. Inclusion criteria were: (1) age approximately 30–80 years, in good health, with no signs or symptoms of any neurological disease; (2) no history of diabetes or hypertension, no use of diuretics, antineoplastic drugs, or sleep disorder medications within the past 3 years, and no history of alcohol abuse or mental illness. Exclusion criteria were: (1) women who are planning pregnancy or are pregnant; individuals with fixed dentures or metal dental implants; (2) family history of neurological or genetic diseases; (3) acute cerebral infarction, tumors, or traumatic brain injury; (4) contraindications to MRI examination; (5) unstable vital signs, major organ failure, or cognitive impairment. The details of patient inclusion and subgroup analyses are summarized in Figure 1.

The study was approved by the Ethics Committee of Suzhou Hospital of Traditional Chinese Medicine (Approval No. 2025 Lunyan Approval 019), and informed consent was obtained from all participants or their legal representatives.

### 2.2 Clinical assessments

Hematoma volume was measured on CT scans obtained within three days of the MRI examination using the ABC/2 method. Exclusion criteria for hematoma volume analysis: (1) patients in the chronic stage (> 3 months post-ictus) who had already developed encephalomalacia; (2) patients who did not undergo CT examination due to clinical circumstances. All measurements were performed by one radiologist with over 5 years of work experience.

Hand motor function was evaluated using the Brunnstrom Recovery Stage for the hand (BRS-H), assessed within three days of MRI by a single experienced clinician. The Brunnstrom Recovery Stage is a tool for evaluating post-stroke motor function recovery, which assesses the motor function separately for the upper extremity, hand, and lower extremity. Based on the six stages of motor recovery post-stroke (including flaccidity, associated reactions, and synergy development, etc.), function is classified into levels I to VI.

### 2.3 MRI acquisition

Images were acquired using a Philips 3.0 T MRI scanner (Ingenia CX, Netherlands) with a 16-channel head–neck joint coil. The participants were scanned in the supine position with the head in a neutral position, and an axial imaging plane oriented along the anterior–posterior commissure (AC-PC) line was utilized. Patient data were acquired using either 15-direction or 32-direction DTI protocol. Healthy volunteers data included both the 15-direction and 32-direction protocols. Additionally, all patients and volunteers underwent conventional cranial MRI scans. Detailed acquisition parameters are provided in Table 1.

**Table 1:** MRI acquisition parameters.

Item	TR (ms)	TE (ms)	FOV (mm)	Matrix	Slice thickness (mm)	Slice gap (mm)	NSA	*b* value (mm^2^)
15-direction DTI	2,919	73	190 × 217	76 × 85	2.5	0	2	0/800
32-direction DTI	4,600	83	224 × 224	112 × 110	2	0	2	0/800
T2WI tra	4,500	116	230 × 230	328 × 328	5	1	1	
T1WI tra	2000	20	230 × 230	288 × 198	5	1	1	
T2Flair tra	4,800	350	230 × 221	232 × 223	2.5	−1.25	2	
DWI	2,887	79	230 × 230	152 × 106	5	1	1	1,000

Summary of scanning parameters for conventional MRI and DTI sequences, including repetition time (TR), echo time (TE), field of view (FOV), matrix size, slice thickness, slice gap, number of signal averages (NSA), and *b*-value.

### 2.4 ALPS index calculation

The DTI data were further processed through the FMRIB Software Library (FSL, v6.0.1, http://www.fmrib.ox.ac.uk/fsl) pipeline. The processing steps were similar to the steps shown in reference (16) and are stated below.

**Figure 2:**
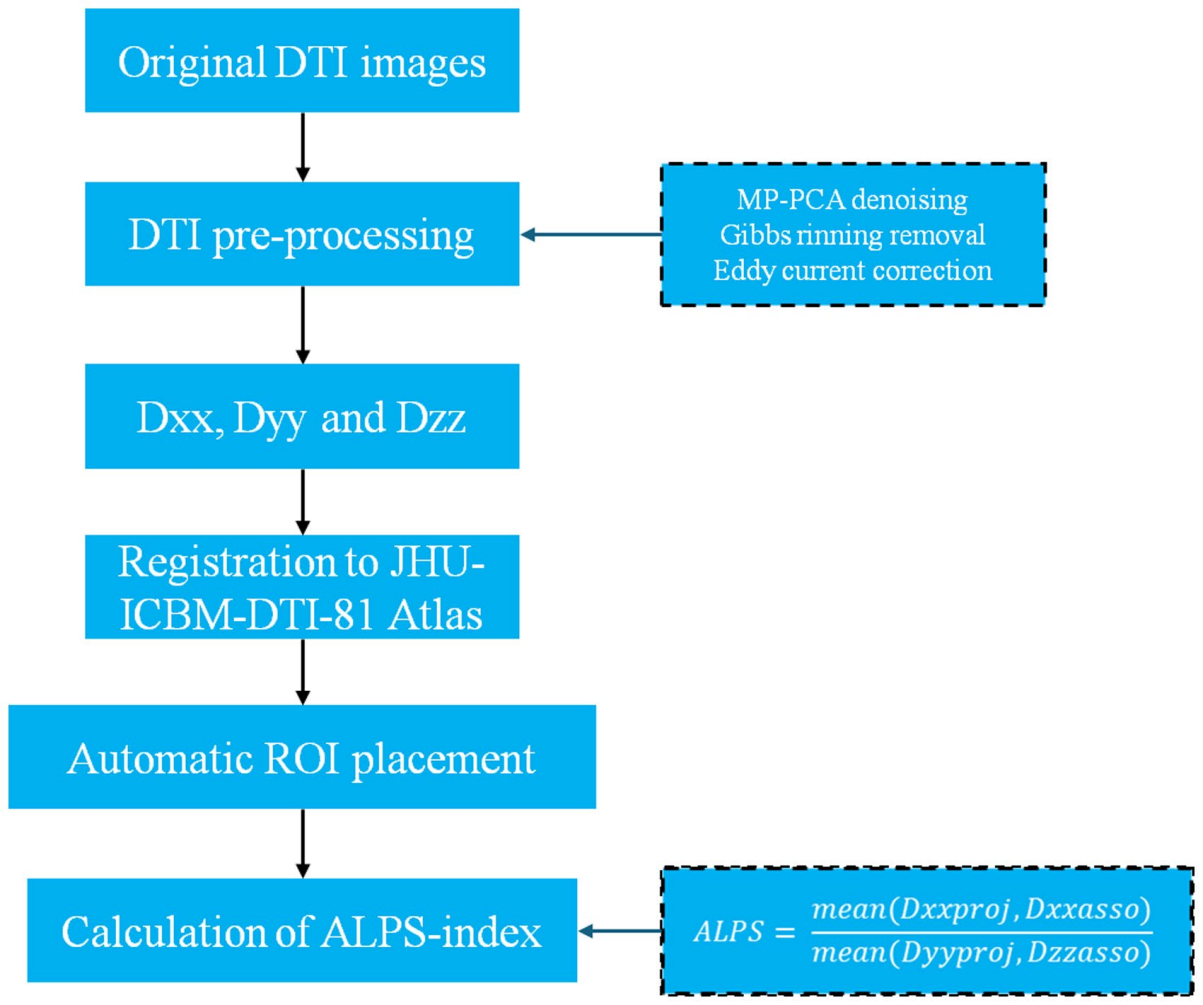
DTI-ALPS processing pipeline DTI-ALPS index calculation workflow. Preprocessing steps include denoising, Gibbs ringing removal, and distortion correction. Diffusivity maps (Dxx, Dyy, Dzz) were generated. Images were nonlinearly registered to MNI space and parcellated using the JHU-ICBM-DTI-81 atlas. ROIs were placed on projection and association fibers in both hemispheres. The ALPS index was calculated as [mean(Dxx_proj + Dxx_assoc)] / [mean(Dyy_proj + Dzz_assoc)].

The original DTI images were preprocessed with denoising, Gibbs ringing removal and distortion correction. Then diffusivity maps (Dxx, Dyy, Dzz) and FA maps were generated. Images were registered to the standard JHU-ICBM-DTI-81-whitematter Labeled Atlasin the MNI space. Four spherical ROIs with 5 mm diameter were defined on the JHU-ICBM-FA template on the projection and association fibers on the left and right hemispheres of the brain. The post-processing flowchart is shown in Figure 2.

The ALPS index was calculated with:


$$\text{ALPS index}=[\begin{array}{l} \text{mean}(\text{Dxxproj}+\text{Dxxassoc})\\ /\text{mean}(\text{Dyyproj}+\text{Dzzassoc}) \end{array}]$$

### 2.5 Statistical analysis

Statistical analysis was conducted using SPSS 25.0 software, and *p* < 0.05 was considered statistically significant. Normality was assessed using the Shapiro–Wilk test on paired differences. Normally distributed data are presented as mean ± standard deviation (SD), while non-normally distributed data are presented as median (interquartile range).

For Healthy Control, Bonferroni correction was applied to control for type I error inflation. The comparisons were divided into two groups: Group 1 (agreement between the two DTI protocols, 3 comparisons, *α* = 0.0167) and Group 2 (left–right hemisphere asymmetry, 2 comparisons, α = 0.025). Paired t-tests were used for normally distributed data and Wilcoxon signed-rank tests for non-normally distributed data. Significance was defined as corrected P < corresponding α. The consistency of ALPS index between DTI-15 and DTI-32 direction was evaluated using intraclass correlation coefficient (ICC) and Bland–Altman plots. The ICC method adopts a bidirectional random effects model, considering single measures and absolute consistency form. According to the criteria by Koo and Li (17): ICC ≤ 0.5: poor consistency; ICC 0.5–0.75: moderate consistency; ICC 0.75–0.9: good consistency; ICC > 0.9: excellent consistency.

Differences in baseline characteristics between the patient and healthy control group, as well as among patient subgroups, were evaluated using Fisher’s exact test or non-parametric tests as appropriate. ALPS index between patients and controls were assessed by independent samples t-test. Among patient subgroups stratified by post-hemorrhage interval (≤ 14 days, 15 days-3 months, 3 months-1 year), an analysis of covariance (ANCOVA) was employed adjusting for age, sex, hypertension, diabetes, scanning protocol, and hemorrhage laterality. As the scanning protocol showed no significant effect on ALPS index across the three patient subgroups (*p* > 0.05), the average of the two ALPS index was used in subsequent analyses. Pairwise comparisons were conducted with LSD correction. Correlations between ALPS index and hematoma volume or BRS-H were examined using Spearman’s rank correlation. Bootstrap resampling (1,000 iterations) was performed to calculate 95% confidence intervals (CIs), and leave-one-out sensitivity analysis was conducted to evaluate the influence of individual cases.

## 3 Results

### 3.1 Healthy control group: protocol comparison

**Figure 3:**
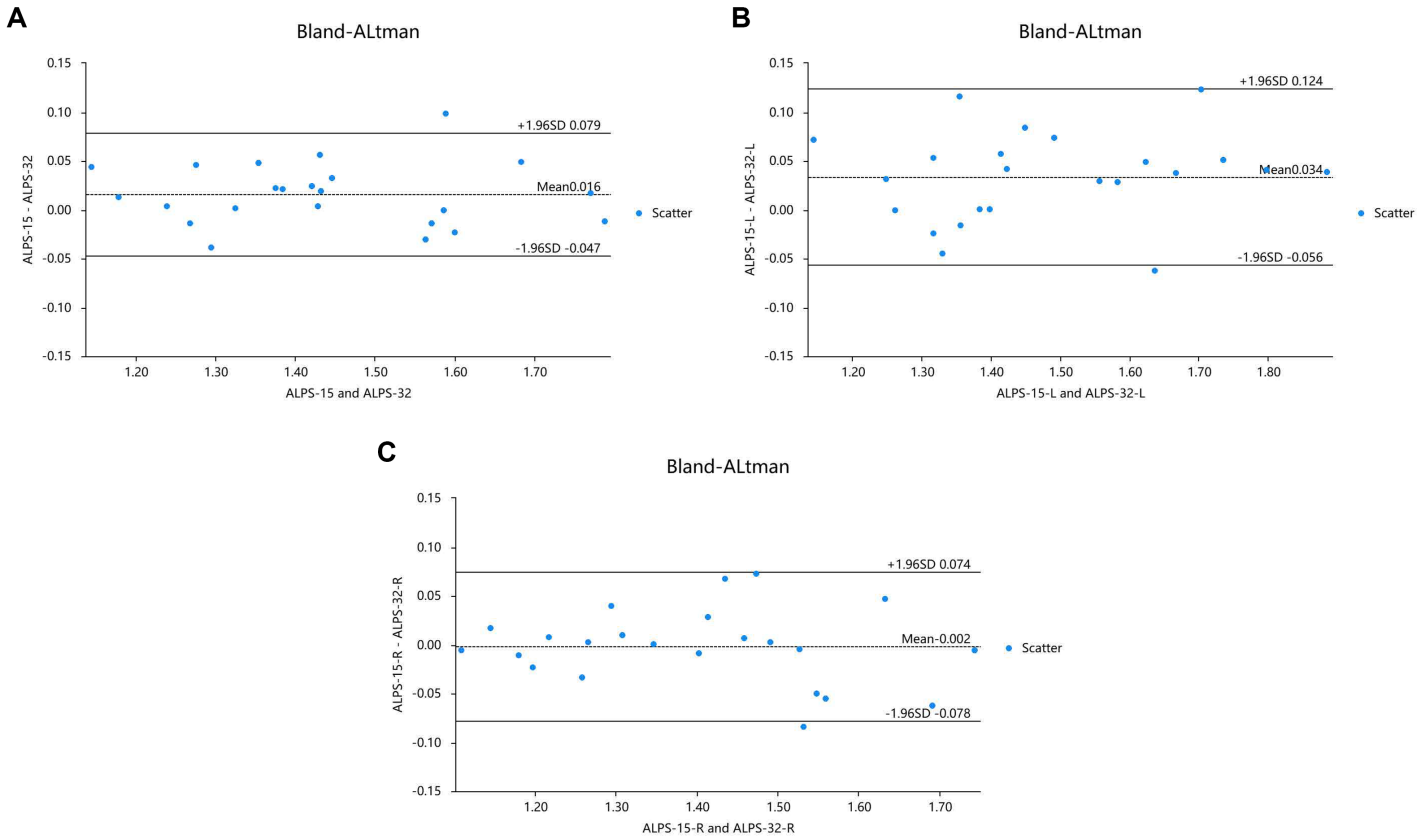
Bland–Altman plots for agreement of ALPS index between 15- and 32-direction DTI protocols in healthy controls. The figure consists of three panels: **(A)** ALPS-mean, **(B)** ALPS-L, and **(C)** ALPS-R. In each panel, the *x*-axis represents the mean of the measurements obtained by the two methods, and the *y*-axis represents the difference between the two measurements. The central dashed line (mean difference) indicates the average of the differences across all data points. The upper and lower solid lines represent the 95% limits of agreement (mean difference ± 1.96 × SD). The vast majority of data points fall randomly within the 95% limits of agreement, indicating that the data obtained from the two DTI protocols have narrow limits of agreement and minimal systematic bias, supporting the comparability of data acquired with different gradient schemes. Notably, the 95% limit of agreement for ALPS-R was the narrowest among all indices.

Normality was violated for ALPS-15 left vs. right (Shapiro–Wilk, *p* = 0.046), thus Wilcoxon test was used; all other paired differences were normally distributed (*p* > 0.05), thus paired t-tests were used. After Bonferroni correction, in Group 1 (protocol agreement), a significant difference was found between the 15-direction and 32-direction protocols in the left hemisphere (corrected *p* = 0.006), while no significant differences were observed for the bilateral mean (corrected *p* = 0.075) or the right hemisphere (corrected *p* = 1.000). In Group 2 (left–right hemisphere asymmetry), significant differences between the left and right hemispheres were found within both the ALPS-15 and ALPS-32 protocols (corrected *p* = 0.004 and 0.018, respectively). The results of the healthy control group are shown in Tables 2, 3, and Figure 3. Interhemispheric comparisons revealed significantly higher ALPS index on the left than the right in both protocols (*p* < 0.01). ICC analysis demonstrated excellent consistency between the two protocols for all measures (ICC range: 0.959–0.981, *p* < 0.001). Bland–Altman plots showed narrow limits of agreement and minimal systematic bias, supporting the comparability of data acquired with different gradient schemes.

**Table 2:** Comparison of ALPS INDEX BETWEEN 15- and 32-direction DTI protocols in healthy controls.

Comparison items	Mean ± SD/Median (IQR)	*p*-value
L: ALPS-15 vs. ALPS-32	1.50 ± 0.20 vs. 1.46 ± 0.19	0.006^*^
R: ALPS-15 vs. ALPS-32	1.40 ± 0.17 vs. 1.40 ± 0.18	1.000
mean: ALPS-15 vs. ALPS-32	1.45 ± 0.18 vs. 1.43 ± 0.18	0.075
ALPS-15: L vs. R	1.44 (1.34–1.65) vs. 1.43 (1.24–1.52)	0.004^*^
ALPS-32: L vs. R	1.46 ± 0.19 vs. 1.40 ± 0.18	0.018^*^

*Statistically significant after Bonferroni correction.

ALPS-15: ALPS index derived from DTI data acquired with 15 diffusion directions.

ALPS-32: ALPS index derived from DTI data acquired with 32 diffusion directions.

mean: Average value of left and right hemispheres.

L: left cerebral hemisphere.

R: right cerebral hemisphere.

**Table 3:** Intraclass correlation coefficient analysis of ALPS index between DTI protocols.

Comparison items	ICC	95%CI	*p*-value
ALPS-15 VS ALPS-32 mean	0.981	[0.947; 0.992]	<0.001
ALPS-15 VS ALPS-32 L	0.959	[0.829; 0.986]	<0.001
ALPS-15 VS ALPS-32 R	0.977	[0.947; 0.990]	<0.001

### 3.2 Patient characteristics

A total of 56 patients were included, with disease duration ranging from approximately 3 days to 1 year (the time of hemorrhage was determined based on clinical history records) and hematoma volumes ranging from 4.2 to 44.5 mL. Based on previous literature (18, 19), patients were divided into three groups according to the time since hemorrhage: Group A (*n* = 34): ≤ 14 days; Group B (*n* = 12): 15 days to 3 months; and Group C (*n* = 10): 3 months to 1 year. No significant differences were observed among groups in age, sex, hypertension, diabetes, or BRS-H (*p* > 0.05). Compared to healthy controls, patients showed no significant differences in age or sex distribution. Detailed results are presented in Table 4.

**Table 4:** Baseline clinical characteristics of study participants.

Characteristics	Control	Patients			
		Group 1	Group 2	Group 3	*p*-value
Age, Mean ± SD	36–76 (55.83 ± 11.16)	32–91 (59.88 ± 13.41)	32–76 (53.83 ± 12.77)	55–77 (65.60 ± 7.37)	0.085
Woman, *n* (%)	7 (30.43%)	10 (29.41%)	5 (41.67%)	3 (30.00%)	0.725
Hypertension, *n* (%)	—*^a^*	26 (76.47%)	6 (50.00%)	7 (70.00%)	0.194
Diabetes, *n* (%)	—	5 (14.70%)	1 (8.33%)	3 (30.00%)	0.451
BRS-H, *n* Median (IQR)	—	33 4 (3–5)	11 2 (1–6)	7 4 (3–5)	0.838

Mean ± SD: Normally distributed data are presented as mean ± standard deviation.

^a^Patients without this classification are indicated by —.

BRS-H, Brunnstrom Recovery Stage for the Hand.

Median (IQR): Non-normally distributed data are presented as median (interquartile range, IQR).

### 3.3 ALPS index in patients vs. controls and across time points

The ALPS index in the healthy control group (mean of ALPS-15 and ALPS-32: 1.44 ± 0.18) was significantly higher than that in the overall patient group (1.21 ± 0.13, *p* < 0.001). Within the patient group, after controlling for the effects of sex, age, hypertension, diabetes, the scanning protocol and hemorrhage location, ANCOVA revealed a significant difference in ALPS index among the three groups (*p* = 0.038). Notably, the interaction between group and scanning protocol (15-direction vs. 32-direction) was not significant (*p* > 0.05), therefore, the average of the two ALPS index was used in subsequent patient group analyses. (Note: Interaction terms between group and scanning protocol, as well as between group and hemorrhage location, were also not significant, *p* = 0.894 and 0.601, respectively, and thus not included in the final model.). Post-hoc pairwise comparisons with LSD correction indicated a significant difference between Groups A and B (*p* = 0.016), while no statistically significant differences were observed between Groups A and C (*p* = 0.155) or between Groups B and C (*p* = 0.487). The adjusted ALPS index were as follows: Group A: 1.24 ± 0.03; Group B: 1.14 ± 0.04; Group C: 1.18 ± 0.04.

A total of seven follow-up assessments were completed among the six patients with longitudinal follow-up. Regarding change trends: three follow-up assessments indicated a persistent decline in the ALPS index within 3 months; three assessments showed a rebound in the ALPS index between 3 months and 1 year, with one assessment demonstrating ALPS index values that even exceeded the level measured within 2 weeks post-bleed. However, one assessment from a patient at 5 months still showed an ALPS index lower than that on day 7 post-bleed. Detailed follow-up data for the six patients are presented in Table 5.

**Table 5:** Longitudinal follow-up timeline for six patients (non-uniform, real-world data).

Patient ID	0–14 days (ALPS index)	15–90 days (ALPS index)	91–365 days (ALPS index)
p01	—	17 (1.143)	96 (1.289)
p03	4 (1.286)	19 (1.195)	—
p04	4 (1.195)	19 (1.186)	120 (1.223)
p05	—	25 (1.111)	365 (1.121)
p06	4 (1.241)	83 (1.157)	—
p07	7 (0.976)	—	146 (0.994)

Values are presented to three decimal places to preserve individual variability; all other data in the manuscript are reported to two decimal places.

— indicates that no MRI examination was performed during the corresponding time window.

### 3.4 Correlation with hematoma volume

Of the 56 patients, 10 were excluded due to chronic-stage encephalomalacia and 4 were excluded because CT data were missing. Ultimately, 42 patients were included in the correlation analysis. Based on hematoma volume, these 42 patients were divided into two groups: 26 patients with hematoma volume ≤ 10 mL and 16 patients with hematoma volume > 10 mL.

No significant correlation was observed between ALPS index and hematoma volume in the overall analysis (*r* = −0.248, *p* = 0.128). However, subgroup analysis revealed a significant negative correlation in the ≤ 10 mL group (*r* = −0.529, *p* = 0.005, *n* = 26), while no significant correlation was found in the > 10 mL group (*r* = −0.091, *p* = 0.737, *n* = 16). Sensitivity analysis: Bootstrap 95% CIs were [−0.776, −0.215] for the ≤10 mL subgroup (excluding zero) and [−0.560, 0.402] for the > 10 mL subgroup (including zero). Leave-one-out analysis yielded *ρ* ranges of −0.518 to −0.551 and −0.18 to −0.01, respectively, with no change in significance. ANCOVA showed no statistically significant difference in ALPS index between the two subgroups (*p* = 0.443).

### 3.5 Correlation with BRS-H

No significant correlation was observed between BRS-H and the ALPS index (*r* = 0.219, *p* = 0.123, *n* = 51), data were missing for 5 patients due to incomplete clinical records).

## 4 Discussion

### 4.1 Robustness of the DTI-ALPS index

To evaluate the robustness of the ALPS index under different acquisition protocols, we enrolled 23 healthy volunteers who underwent two DTI acquisition protocols were performed on the same 3.0 T MRI scanner: one with 15 non-collinear diffusion encoding directions and the other with 32 directions. To eliminate subjective bias in ROI selection and obtain accurate and highly reproducible ALPS index, an automated post-processing technique was employed (20, 21). The comparison of DTI protocols in healthy controls revealed systematic difference in ALPS index between 15- and 32-direction acquisitions exclusively in the left hemisphere. Indicate that under the current scanning parameters, there is a small but consistent systematic offset in ALPS-L values between the 15-direction and 32-direction protocols. The high consistency between the two datasets supports the comparability of results across different gradient schemes. This discrepancy may be attributable to the more complex microstructural architecture of the left hemisphere. This hypothesis is supported by both previous literature (20) and the observed interhemispheric differences in our study, wherein the ALPS index in the left hemisphere was significantly higher than that in the right hemisphere (*p* < 0.05).

Neurobiological evidence indicates that radial asymmetry is a ubiquitous feature of white matter axonal organization (22, 23). Crossing fibers contribute to this radial asymmetry and have been shown to significantly inflate the ALPS index (22). Consequently, the ALPS index may reflect a combined effect of fiber radial asymmetry and glymphatic diffusion, and may also be influenced by other factors such as the size, shape, and placement of regions of interest (ROIs) (24, 25), based on this theoretical framework, the 32-direction DTI protocol in our study employed a thinner slice thickness, which theoretically yields higher image resolution and clearer fiber visualization. Moreover, a greater number of diffusion gradient directions enables more precise estimation of the diffusion ellipsoid shape, more accurate delineation of fiber orientations, and improved resolution of complex crossing fibers. Consequently, the ALPS index derived from the 32-direction DTI protocol would be higher. Contrary to theoretical predictions and prior reports (24), the ALPS index obtained from the 32-direction DTI protocol was significantly lower than that from the 15-direction protocol in our study (*p* < 0.05). It is likely that radial asymmetry of white matter fibers contributes only modestly to the ALPS index. Taoka et al. reported that shorter echo time (TE) is associated with higher ALPS index (24). Consistent with this finding, our 15-direction protocol (TE = 73 ms) produced higher ALPS values than the 32-direction protocol (TE = 83 ms). Therefore, the TE difference likely exerts a stronger influence on the ALPS index and may account for the discrepancy between the two protocols. Furthermore, other study demonstrated no significant interhemispheric difference in the ALPS index (25). This finding, together with our observations, suggests that the ALPS index is influenced by a multitude of factors, and further studies are required to elucidate its determinants.

In conclusion, despite a small systematic bias (ALPS-15 > ALPS-32) between the two DTI protocols, the data exhibited excellent consistency.

### 4.2 Effect of basal ganglia hemorrhage on the ALPS index

In this study, the ALPS index was significantly decreased in patients with basal ganglia hemorrhage relative to healthy controls matched for age and sex (*p* < 0.001). Indicates that ICH is associated with GS dysfunction (9, 15, 26, 27). ICH impairs the GS via multiple mechanisms. Initially, direct injury to small vessels and perivascular spaces occurs at the hemorrhage site. Subsequently, mass effect from the hematoma compresses the perivascular spaces, obstructing glymphatic drainage (28, 29) and resulting in decreased ALPS index. With hematoma resolution, erythrocyte lysis liberates hemoglobin and other toxic byproducts, triggering the release of pro-inflammatory cytokines (5, 30). This neuroinflammatory response, in turn, compromises glymphatic function by degrading the perivascular basement membrane and disrupting AQP-4 polarization (31). Impairment of the GS causes accumulation of soluble amyloid-*β* (Aβ) (7), ro-inflammatory cytokines, and neurotoxic substances, which ultimately compromises the blood–brain barrier (BBB) (32).

Evidence from prior stroke research suggests that spontaneous recovery initiates within 1–2 weeks in animal models and extends over a longer period in humans (18), with the most substantial motor gains observed in the first three months (33). Since the lesions in our study predominantly involved the basal ganglia, a region critical for limb motor function, we adopted 14 days and 3 months as temporal thresholds for patient stratification. Statistical analysis revealed that: (1) Compared with the first 14 days, the ALPS index showed a significant decrease during the 14-day to 3-month period (*p* < 0.05), which appears contrary to theoretical predictions (33). Theoretical considerations would suggest that the motor recovery phase between 2 weeks and 3 months post-stroke might be associated with neurovascular and glymphatic remodeling, potentially leading to an elevated ALPS index. However, we observed a sustained decrease. One possible interpretation is that neuroinflammatory damage may have predominated over glymphatic reconstruction during this period. Specifically, persistent neuroinflammation might have offset the modest increase in ALPS index that could otherwise be expected from partial glymphatic remodeling associated with motor recovery, resulting in the net decrease observed between 14 days and 3 months. This proposal is supported by temporal profiles of post-ICH inflammation and AQP-4 depolarization. In early ICH, activated inflammatory cells disrupt the BBB, allowing peripheral immune cells to infiltrate the brain. While most return to baseline within 14 days, NK cells persist up to four weeks, creating a sustained neuroinflammatory microenvironment that impairs microvascular maturation and drives continued ALPS decline (34, 35). Concurrently, AQP-4 depolarization peaks at day 3 and causes glymphatic dysfunction (36), although partial recovery occurs (37), significant depolarization remains detectable at day 14 (38), indicating persistently reduced glymphatic function. Together, these mechanisms explain why neuroinflammation-driven glymphatic impairment persists during the 14-day to 3-month window, accounting for the observed sustained decrease in the ALPS index. It should be noted that Zhang et al. (15) reported an increase in the ALPS index one month after subarachnoid hemorrhage, which is not entirely consistent with our findings. This discrepancy might be explained by the possibility that subarachnoid hemorrhage primarily affects meningeal lymphatic drainage (39), which may not directly parallel changes in the GS. (2) From 3 months to 1 year, the ALPS index increased but did not reach the level observed within the first 14 days. This phenomenon may be explained by two potential mechanisms. First, it is possible that the GS in the perilesional area reaches a state of functional exhaustion by 3 months, with the ALPS index declining to its minimum. During the chronic stage, residual hemosiderin and ongoing neuroinflammation (40) may establish a persistently unfavorable microenvironment, perpetuating glymphatic injury through sustained disruption of AQP-4 polarity and perivascular fibrosis (41), potentially leading to progressive glymphatic dysfunction. Longitudinal studies on ICH have shown that CBF in the hematoma and surrounding tissues remains consistently and significantly reduced compared with the contralateral hemisphere throughout the post-hemorrhage course (6), which may support the presence of sustained glymphatic impairment in the perilesional area. A second potential explanation relates to the prolonged time course of functional recovery. Improvements in cognition, memory, and language may continue for months to years after stroke (33), potentially accompanied by sustained neurovascular remodeling and progressive glymphatic recovery. Additionally, extensive growth of meningeal lymphatic vessels—the downstream component of the glymphatic pathway—may occur (42), and these factors together might contribute to the delayed increase in the ALPS index. Furthermore, it is worth noting that the patient group in this study had a wide age range, and most patients were older. Given that age is known to influence glymphatic function and neuroplasticity, this may have further contributed to the delayed glymphatic recovery observed in this cohort. Our findings are partially consistent with those of Chen et al. (19), who reported an increase in the ALPS index over time after ischemic stroke. The discrepancy between our results and theirs might be explained by differences in stroke types (ischemic vs. hemorrhagic) and lesion locations (lobar vs. deep basal ganglia), which may involve distinct pathophysiological mechanisms. Xia et al. (26) found no significant change in the ALPS index at three weeks post-hemorrhage. This inconsistency may be attributable to variations in grouping strategies, interventions, sample characteristics, and lesion locations. Larger-scale longitudinal studies are warranted to further clarify these issues.

### 4.3 Correlation between ALPS index and clinical parameters

Basal ganglia hemorrhage frequently involves the corticospinal tract and the corticocerebellar tract, both of which are important neural pathways for motor function regulation. Hand motor function is a core indicator for assessing patients’ activities of daily living and quality of life. Accordingly, this study focused on analyzing the correlation between the stages of hand motor recovery and the ALPS index, aiming to explore the potential role of GS function in motor rehabilitation. The results of this study revealed no significant correlation between the ALPS index and BRS-H. Several factors may account for this finding. First, as a motor function assessment tool, BRS-H employs relatively broad categorical grading, which may lack the sensitivity to capture subtle variations in the ALPS index. The ALPS index, a continuous variable reflecting GS function, exhibits high sensitivity to changes in neurological status (19), whereas the discrete nature of BRS-H grading may dilute the potential association between the two. Second, although basal ganglia hemorrhage predominantly presents with motor dysfunction, affected patients frequently exhibit concurrent impairments involving multiple ascending and descending brain regions, manifesting as deficits in language, emotion, cognition, and other domains. Neurological recovery is a complex biological process that relies on interactions, functional reorganization, and compensation among distributed brain networks. Therefore, as an imaging biomarker reflecting global glymphatic function, dynamic changes in the ALPS index may comprehensively capture the integrated effects of pathophysiological processes across multiple brain regions, rather than exclusively mirroring motor recovery. This further explains the absence of a significant correlation between the ALPS index and a single motor function assessment tool.

In the overall cohort of this study, no significant correlation was observed between hematoma volume and the ALPS index. Stratified analysis revealed a significant negative correlation between hematoma volume and the ALPS index in the ≤ 10 mL subgroup, whereas not in the > 10 mL subgroup. Possibly because the lesions in this cohort were predominantly located in the basal ganglia region, an area rich in ascending and descending nerve fibers and dense perivascular spaces. Even a small hematoma may induce significant local alterations in the glymphatic pathway, manifesting as notable changes in the ALPS index. In contrast, when hematoma volume exceeds a certain threshold (e.g., 10 mL in the present cohort), the pattern of GS impairment may shift from predominantly local (perivascular space compression and focal perivascular pathway disruption) to predominantly global (widespread BBB breakdown, impaired meningeal lymphatic drainage, and dysfunction of arachnoid granulations). Aksoy et al. (43) used dynamic contrast-enhanced MRI to analyze the relationship between hematoma volume and BBB permeability. They found that the BBB permeability (volume transfer constant, Ktrans) was significantly higher in large hematomas (≥ 30 mL) than in small hematomas, but the range of Ktrans values was substantially wider in the large hematoma group compared to the narrow range in the small hematoma group. This finding suggests that BBB permeability does not increase proportionally with hematoma volume in the large hematoma group, implying that when hematoma volume reaches a certain threshold, further increases in volume no longer linearly exacerbate BBB disruption. Although no similar study has investigated the relationship between hematoma volume and the GS, given that the GS and the BBB share perivascular spaces and astrocytic endfeet, we speculate that the GS may exhibit a similar pattern. Furthermore, due to remote dissemination of neuroinflammation and other factors, a larger hematoma volume may cause global GS impairment, thereby attenuating the correlation between larger hematoma volume and local glymphatic function. It should be noted that the ALPS index only assesses local glymphatic function. When hematoma volume exceeds a threshold, global glymphatic impairment may no longer be effectively captured by the ALPS index. Under such circumstances, the ALPS index may no longer correlate solely with the local hematoma volume but instead reflect the functional status of the global GS, which may explain the absence of a significant correlation between larger hematoma volume and the ALPS index. Previous studies have suggested the existence of a prognostic threshold for supratentorial intraparenchymal hematoma volume: patients with hematoma volume below this threshold tend to have favorable outcomes, whereas those exceeding the threshold often experience poor prognosis (44). Although the specific role of the GS in supratentorial deep ICH remains to be fully elucidated, and targeted therapies directed at the GS have not yet been widely implemented in clinical practice, accumulating evidence indicates that glymphatic function is closely associated with the prognosis of ICH. Therefore, the impact of the GS should be considered in the clinical management of supratentorial deep hematomas. Notably, previous studies have reported a significant correlation between the ALPS index and hematoma volume (9, 26), which is not entirely consistent with the findings of the present study. This discrepancy may be attributed to differences in study population composition: prior studies included patients with various types of hemorrhage, including supratentorial and infratentorial bleeds, which may limit the applicability of their conclusions to specific anatomical locations. Additionally, the relatively small number of cases with larger hematoma volumes in this cohort may have constrained the statistical power of this subgroup analysis to some extent. The clinical significance of this stratified analysis lies in its consistency with the established pattern of hematoma volume on prognosis: within a specific volume threshold, interventions targeting the GS may contribute to improved patient outcomes; whereas when the hematoma volume exceeds the threshold, more aggressive therapeutic strategies may be required to address global cerebral injury, such as BBB disruption.

## 5 Limitations

Several limitations of this study should be acknowledged. First, the composition of the study cohort changed during the follow-up period, leading to heterogeneity among samples at different time points. Therefore, the observed differences may be partially attributable to variations in sample characteristics rather than purely longitudinal effects, warranting caution in causal inferences. Future studies should establish strict inclusion criteria and, whenever possible, conduct complete longitudinal follow-up. Second, the sample size of this study was relatively small, which limited our ability to perform multivariate analyses. The sample size of subgroup C was particularly small (*n* = 10), and the > 10 mL subgroup (*n* = 16) showed wide bootstrap CIs, indicating limited precision. Further validation in larger prospective cohorts is warranted. Third, although age was adjusted for in ANCOVA and showed no significant across-subgroup difference, residual confounding by age cannot be excluded. Stricter age matching is needed in future studies. Fourth, we did not systematically assess the severity of chronic cerebrovascular disease, which may have confounded the results. Future studies should quantify this burden and include it as a covariate. Fifth, this exploratory study identified a significant correlation between the ALPS index and hematoma volume in patients with hematoma volume < 10 mL. However, sensitivity analyses using alternative cutoffs (detailed results are presented in Supplementary Table S1) yielded inconsistent results, warranting validation in larger prospective cohorts.

## 6 Conclusion

This study demonstrates that basal ganglia hemorrhage can induce persistent GS injury, and that delayed recovery of glymphatic function may be associated with multisystem functional recovery. Targeting GS function may offer a novel therapeutic strategy for ICH (45).

## Data Availability

The raw data supporting the conclusions of this article will be made available by the authors, without undue reservation.
