# Supplementary material for: Longitudinal changes in the ALPS index and its clinical correlates in patients with basal ganglia hemorrhage
Source: Front Neurol. 2026 Jul 9;17:1839634. doi: 10.3389/fneur.2026.1839634 (PMC13391268; doi:10.3389/fneur.2026.1839634)
Supplement: Supplementary file 1 [file Table_1.DOCX]

**Supplementary Table S1. Sensitivity analyses for the correlation between ALPS index and hematoma volume using alternative cutoffs**

| Sensitivity analysis | Group | n | Correlation coefficient (r) | P value |
| --- | --- | --- | --- | --- |
| 5ml cutoff | <5 mL | 13 | -0.364 | 0.222 |
|  | ≥5 mL | 29 | 0.016 | 0.936 |
| 10ml cutoff (primary) | <10 mL | 26 | -0.529 | 0.005 |
|  | ≥10 mL | 16 | -0.091 | 0.737 |
| 15ml cutoff | <15 mL | 32 | -0.297 | 0.099 |
|  | ≥15 mL | 10 | -0.188 | 0.603 |
| Median split (6.7ml) | <6.7 mL | 20 | -0.344 | 0.15 |
|  | ≥6.7 mL | 22 | 0.172 | 0.445 |
